# Protection from within

**DOI:** 10.7554/eLife.24111

**Published:** 2017-01-12

**Authors:** Florent Masson, Bruno Lemaitre

**Affiliations:** Global Health Institute, School of Life Sciences, École Polytechnique Fédérale Lausanne, Lausanne, Switzerland; Global Health Institute, School of Life Sciences, École Polytechnique Fédérale Lausanne, Lausanne, Switzerlandbruno.lemaitre@epfl.ch

**Keywords:** tsetse fly, symbiont, odorant binding protein, hematopoiesis, crystal cell, symbiosis, *D. melanogaster*

## Abstract

The development of the tsetse fly immune system relies on a cue from an endosymbiotic bacterium called *Wigglesworthia.*

**Related research article** Benoit JB, Vigneron A, Broderick NA, Wu Y, Sun JS, Carlson JR, Aksoy S, Weiss BL. 2017. Symbiont-induced odorant binding proteins mediate insect host hematopoiesis. *eLife* **6**:e19535. doi: 10.7554/eLife.19535

Symbiotic interactions between eukaryotes and microbes can result in a wide variety of phenotypes for the host. These interactions can, for example, help the host to colonize habitats in which relatively little nutrition is available or to adapt to extreme environments (Douglas, 2015; McFall-Ngai et al., 2013). Symbiotic interactions with microbes can also offer "protection" to eukaryotes by helping them to resist their natural enemies. Many examples of the protection phenotype have been observed and, in recent years, researchers have started to unravel the mechanisms that underpin symbiosis-mediated protection.

Some of these examples involve a direct interaction between the microbe and pathogens that threaten the host (see Table 1). For example, some microbes produce toxins that can inhibit the growth of (or even kill) a specific species of pathogen (Hamilton et al., 2016), while others produce chemicals that target a range of pathogens (Gil-Turnes et al., 1989; Cirimotich et al., 2011). Symbionts can also offer protection by occupying niches in the host that the pathogen would otherwise occupy. For example, it is thought that the presence of large numbers of microbes in the gut (the gut microbiota) prevents it being colonized by opportunist pathogens in mammals. This would explain the increased frequency of opportunistic infection following an antibiotic treatment (Bignardi, 1998).

**Table 1.** Mechanisms of symbiosis-mediated protection.

		Mechanism	Description	Examples*
Direct interaction		Toxin synthesis	The symbiont produces a toxin that is harmful to parasites	*-* The endosymbiont *Spiroplasma* protects *Drosophila* against nematodes by producing RIP toxins (Hamilton et al., 2016) - Bacteria protect aphids against parasitoid wasps by releasing a phage that kills the wasp larvae (Oliver et al., 2009) *-* The gut microbiota generates reactive oxygen species that protect *Anopheles* mosquitoes against infection by *Plasmodium* parasites (Cirimotich et al., 2011)
		Niche colonization	The symbiont and the parasite compete for the same space	- The gut microbiota prevents the colonization of the digestive tract by opportunistic bacteria (as suggested by the frequent colonization of the human gut by *Clostridium difficile* upon antibiotic treatment: see, for example, Bignardi, 1998). - Members of native coral microbiota inhibit the colonization of coral mucus by opportunistic pathogens (Krediet et al., 2013)
Indirect interaction	Immune-related	Immune system development	The symbiont triggers the maturation of the host immune system during development	- The endosymbiont *Wigglesworthia* stimulates hematopoiesis in its host the tsetse fly (Weiss et al., 2011; Benoit et al., 2017) - The gut microbiota stimulates intestinal lymphoid tissue maturation in some mammals (see, for example, Gaboriau-Routhiau et al., 2011)
		Immune system activation	The symbiont enhances an immune response that prevents infection by a parasite	- The gut microbiota stimulates the immune system to maintain a basal level of immune defense, or to increase the immune reactivity, in insects, mammals (see, for example, Broderick et al., 2014)
	Metabolism-related	Metabolic competition	The symbiont consumes host-supplied resources that are also needed by the parasite	- The endosymbiont *Wolbachia* protects mosquitoes against viruses by competing for lipid (Caragata et al., 2013) *-* The endosymbiont *Spiroplasma* protects *Drosophila* against parasitoid wasps by competing for lipid (Paredes et al., 2016)
		Metabolic or endurance enhancement	The symbiont improves the host's overall physiology or increases endurance to parasites	- Symbiotic bacteria in the intestine stimulate epithelium renewal, which improves the endurance of *Drosophila* against a virus (Sansone et al., 2015) - Symbiotic bacteria supply vitamins and amino acids, which helps their host to survive infection (as suggested by the general weakness of many germ-free raised animals)

* In most cases the precise mechanism is not fully established due to the number and complexity of the interactions involved.

There are also examples of protection that result from indirect interactions between the microbe and pathogens (see Table 1). These indirect interactions can be mediated by the metabolism or immune system of the host. In the case of metabolic competition, the symbiont provides protection by depleting resources (supplied by the host) that the pathogen would normally rely on. These resources are often lipids such as cholesterol or diacylglycerides (Caragata et al., 2013; Paredes et al., 2016).

Symbiotic bacteria in the intestine can also provide protection by stimulating the immune system. In *Drosophila*, for example, the gut microbiota can sensitize the host immune system so that it reacts more promptly and efficiently to subsequent infections. And in some cases, such as the lymphoid mucosal immune system in mammals, microbes in the gut are needed to build the mature immune system (Gaboriau-Routhiau et al., 2011). Now, in eLife, Brian Weiss of Yale University and co-workers – including Joshua Benoit of the University of Cincinnati as first author – report the results of experiments on the tsetse fly which show that similar mechanisms are at work in species other than mammals (Benoit et al., 2017).

The tsetse fly is a viviparous insect that is host to an endosymbiotic bacterium called *Wigglesworthia*, which is found inside somatic cells called bacteriocytes and also in the organ that produces milk in female tsetse flies. As the tsetse fly larva develops inside its mother’s uterus, it receives *Wigglesworthia* through the consumption of milk. *Wigglesworthia*-free offspring can be produced for research purposes by treating female tsetse flies with antibiotics, and Weiss and co-workers have shown previously that *Wigglesworthia*-free adult flies have a reduced number of cells called hemocytes, which leads to a decreased resistance to infections (Weiss et al., 2011). Benoit et al. have now shown that a protein called Obp6 (short for odorant binding protein 6) is a key player in this process.

Hemocytes have a central role in the immune system of invertebrates, and they are usually found in a fluid called the hemolymph (which is analogous to the blood of vertebrates). Benoit et al. now show that the presence of *Wigglesworthia* leads to the production of Obp6. They also show that, during development, this protein regulates a number of pathways that are involved in insect hematopoiesis (that is, the proliferation of hemocyte progenitor cells and their differentiation into various subtypes). In particular it is thought that Obp6 drives the differentiation of hemocyte precursor cells into functional hemocyte cells called crystal cells. These cells are involved in the melanization reaction, which involves the deposition of a substance called melanin around pathogens, and they also help to produce reactive oxygen species that can combat microbes. *Wigglesworthia*-free tsetse flies have significantly reduced levels of Obp6, which leads to a lack of crystal cells and a faulty melanization response when they are infected.

Since *Wigglesworthia* also provides essential micronutrients and vitamins to the tsetse fly, a challenge for the future is to differentiate between the protection conferred by an active mechanism (via Obp6 and crystal cells) and the protection that comes from being more healthy and, therefore, probably more able to cope with pathogens (courtesy of the essential metabolites).

Another challenge is to learn more about the evolution of symbiosis-mediated protection in different species. Thus, Benoit et al. also performed experiments on fruit flies, which are different from tsetse flies in that their gut bacteria are not intimately associated with the host (and are not vertically transmitted from generation to generation). The researchers found that fruit flies that have been deprived of their gut bacteria during development are similar to tsetse flies in that they mount a weaker melanization reaction. This suggests that the mechanism that is responsible for the microbe-mediated stimulation of hematopoiesis could be conserved in insects.

It is likely that the continuous presence of bacteria in eukaryotes over the course of evolution has led to the development of an integrated system, where the host relies on signals from its symbionts to build its own physiology. One of the main challenges in understanding symbiont-mediated protection is to identify the signal (or signals) that link the increase in hematopoiesis to the presence of the symbiont response and protection phenotype. This will provide information on the extent to which symbionts actively participate in host protection and shed new light on the molecular dialogue that rules the interactions between microbes and their hosts.
